# Magnetic field induced uniaxial alignment of the lyotropic liquid-crystalline PMMA-grafted Fe_3_O_4_ nanoplates with controllable interparticle interaction†

**DOI:** 10.1039/c9na00767a

**Published:** 2020-01-14

**Authors:** Chen Shen, Masaki Matsubara, Mizuho Yabushita, Sachiko Maki, Atsushi Muramatsu, Kiyoshi Kanie

**Affiliations:** Institute of Multidisciplinary Research for Advanced Material, Tohoku University 2-1-1 Katahira Aoba-ku, Sendai Miyagi 980-8577 Japan kanie@tohoku.ac.jp; National Institute of Technology, Sendai College 48 Nodayama, Medeshima-Shiote Natori Miyagi 981-1239 Japan

## Abstract

Magnetite (Fe_3_O_4_) nanoplates with a hexagonal platelet shape were synthesized by two steps: hydrothermal synthesis of iron(iii) oxide (α-Fe_2_O_3_) nanoplates followed by wet chemical reduction of the α-Fe_2_O_3_ nanoplates. Then, poly(methyl methacrylate) (PMMA) chains were grafted onto the surface of the hexagonal Fe_3_O_4_ nanoplates (F) *via* surface-initiated atom transfer radical polymerization (SI-ATRP), which ensures dispersion stability in organic solvents and ionic liquids. After mixing with 1-ethyl-3-methylimidazolium bis(trifluoromethanesulfonyl)imide ([Emim^+^][NTf_2_^−^]), a representative ionic liquid, the resulting PMMA-modified F were found to show good lyotropic liquid-crystalline (LC) behaviour in [Emim^+^][NTf_2_^−^] and to exhibit a fast response to the application of an external magnetic field. Ultrasmall-angle synchrotron X-ray scattering (USAXS) measurements verified that the PMMA chain length, the weight ratio of the ionic liquid and the external magnetic field could significantly influence the interparticle distance (*I*_D_) of the PMMA-modified F in [Emim^+^][NTf_2_^−^]. In particular, the lyotropic LC phase could be assigned as a nematic phase with a columnar alignment. In addition, the PMMA-modified F maintained a uniaxially aligned nematic columnar structure along the magnetic field direction. Our study also determined the mechanism for the special alignment of the PMMA-modified F under an external magnetic field by analysing the growth axis, the easy magnetic axes, and the interparticle distance of F. The results suggested that the special alignment of the PMMA-modified F was affected by the interparticle interaction caused by the PMMA long chains on F under the magnetic field. Furthermore, the present study revealed that PMMA-modified F exhibited a new magnetic field responsive behaviour that led not only to the formation of a uniaxial alignment structure but also to control of *I*_D_ with the help of the PMMA soft corona under the application of a magnetic field. These features could prove to be a promising advance towards novel applications of magnetic nanoparticles (NPs), such as functional magnetic fluids, rewritable magnetic switching devices, and smart magneto-electrochemical nanosensors.

## Abstract 2

Lyotropic liquid-crystalline PMMA-grafted Fe_3_O_4_ nanoplates with controllable interparticle interaction showed a uniaxially aligned nematic columnar structure under an external magnetic field.

## Graphical abstract

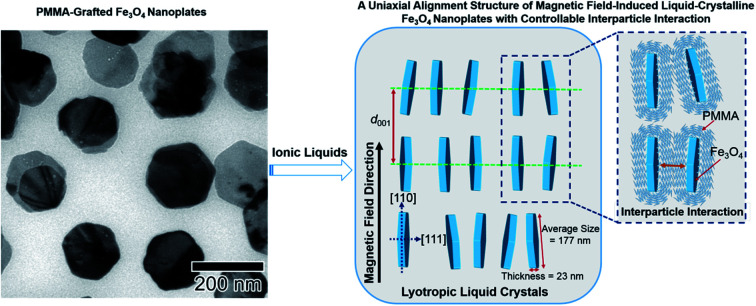

## Introduction

Recent progress in the development of inorganic nanoparticles (NPs) has enabled us to obtain NPs with uniform size, composition, and morphology, which greatly affect the characteristics of the NPs.^1,2^ Over the past decades, magnetic NPs with unique sizes and morphologies have been intensively investigated due to their special physical and chemical properties and various applications.^3,4^ The sizes of magnetic NPs can play a very critical role in the magnetic properties. When the average size of magnetic NPs is the same as the single domain size (∼20 nm), their coercivity can reach the maximum value. The morphologies of magnetic NPs also have a significant influence on the magnetic properties, and controlling the growth of magnetic NPs along the easy magnetic axis is a way to improve the magnetic properties.^5^ In contrast, the magnetocrystalline anisotropy is independent of the grain size and shape as an intrinsic property of magnetic NPs, and the magnetization can reach saturation in different magnetic fields according to the crystallographic orientation of the magnetic NPs.^6^ Bohra *et al.*^7^ reported that the easy [111] and relatively hard [110] axes of magnetization align along texture planes of magnetite (Fe_3_O_4_) NPs under the saturation field.

As representative magnetic NPs, Fe_3_O_4_ NPs with controlled sizes and morphologies have been applied in many fields because of their special electrical properties, biocompatibility, magnetic properties, *etc.*^8–10^ To date, various shaped Fe_3_O_4_ NPs, including nanocubes, nanorods, nanospheres, and nanoplates, have been widely investigated.^11–14^ In particular, hexagonal Fe_3_O_4_ nanoplates with an anisotropic morphology are expected to show marvellous physical and chemical properties. However, Fe_3_O_4_ NPs aggregate easily in fluids due to the interparticle magnetic attractive forces caused by the large surface energy,^15^ which seriously restricts the range of application. However, the process of surface modification for functionalization of inorganic NPs has successfully and rapidly advanced, which has enriched the properties and applications of inorganic NPs.^16–18^ Similarly, it is necessary to combine Fe_3_O_4_ NPs with other modifiers (such as polymer, inorganic, and organic modifiers) to control the interaction and aggregation of NPs.^19–21^ Among the different modifiers, polymer materials have many advantages. The introduction of homogeneous polymer chains on the surface of Fe_3_O_4_ nanoplates promotes variable structure control and synergistic effects of the Fe_3_O_4_ nanoplates and polymers, leading to the expectation of functional fluids. In addition, the dispersion stability of the structured Fe_3_O_4_ nanoplates can be noticeably improved.^22^

One of the most effective ways to prepare polymer-modified NPs is surface-initiated atom transfer radical polymerization (SI-ATRP),^23^ with the advantages of controlling the growth of the polymer chains and improving the modification densities of the polymers. Moreover, SI-ATRP can be applied to a variety of polymers and NPs and can maintain the crystalline structures, sizes, and morphologies of NPs.^24–27^

For some surface-modified inorganic anisotropic particles, their lyotropic liquid-crystalline (LC) properties in colloidal suspensions with uniaxial alignment have been reported.^28,29^ Miyamoto *et al.*^30^ reported that inorganic anisotropic nanosheet colloids showed LC phases with highly ordered lamellar structures. According to our previous research, iron(iii) oxide (α-Fe_2_O_3_) particles and Fe_3_O_4_ particles with anisotropic shapes could be covered by thick polymers using SI-ATRP.^31,32^ The polymer-modified α-Fe_2_O_3_ particles and Fe_3_O_4_ particles exhibited good dispersion stability due to the presence of thick polymers on the surface. In addition, the polymer-modified Fe_3_O_4_ particles, as colloidal fluids based on ionic liquids, showed the lyotropic nematic LC property and a fast response to an external magnetic field.

Among a variety of polymers, poly(methyl methacrylate) (PMMA) has the advantages of high strength, good compatibility, dimensional stability, and optical clarity.^33^ Due to the synergetic effect, PMMA-modified Fe_3_O_4_ NPs are expected to exhibit remarkable properties, such as the lyotropic LC property, superparamagnetism, a low dielectric permittivity, and a high miscibility.^31,34–36^ Furthermore, the PMMA chains on the surface of Fe_3_O_4_ NPs can form a thin film to decrease the aggregation and thus enhance the dispersion stability in some common organic solvents, such as tetrahydrofuran (THF), toluene, and chloroform (CHCl_3_). Thus, PMMA-modified Fe_3_O_4_ NPs are expected to show promise in the exploitation of various functions in magnetorheological and magnetic fluids, electrochemical sensors, and lyotropic liquid crystals (LCs).^37–40^ In particular, magnetorheological fluids (MR fluids), as a type of magnetic suspension whose viscosity increases dramatically in the presence of an external magnetic fields, have potential application in some smart devices.^41^

On the other hand, ionic liquids, as organic solvents, can mix well with PMMA-modified inorganic NPs and exhibit unique rheological and optical properties.^42^ To obtain ionically functional anisotropic materials, ionic liquids need to be mixed with polymer-modified inorganic NPs to form lyotropic LC phases. Crystalline NPs can be conveniently synthesized at ambient temperatures in ionic liquids because of the high polarity and the preorganized solvent structure of the ionic liquids.^43^ Then, new functions of LC materials can be developed using self-organized anisotropic nanostructures.^44^ In fact, ionic liquids have negligible volatility, high ionic conductivity, and high thermal stability compared with traditional solvents, which have stimulated the development of environmentally friendly solvents.^45,46^ As a representative ionic liquid, 1-ethyl-3-methylimidazolium bis(trifluoromethanesulfonyl)imide ([Emim^+^][NTf_2_^−^]) was utilized in this report because of its extremely low vapor pressure and compatibility with PMMA.

In this study, hexagonal Fe_3_O_4_ nanoplates (F) controlled in size and morphology were first synthesized. Then, the monodispersed F were modified by PMMA using SI-ATRP. After grafting thick PMMA chains onto the surface of F, the PMMA-modified F were mixed with [Emim^+^][NTf_2_^−^] in various weight ratios to dissolve the PMMA and form LC phases. Then, the lyotropic LC properties, magnetic response properties and self-organized structures of the PMMA-modified F in [Emim^+^][NTf_2_^−^] were investigated. We also studied the mechanism for the special alignment of the PMMA-modified F under an external magnetic field.

## Experimental

### Materials

All materials (analytic grade) were used as received without further purification.

Iron(iii) chloride hexahydrate (FeCl_3_·6H_2_O), ethanol, sodium acetate, hexylamine, acetonitrile, methanol, *N*,*N*-dimethylacetamide (DMAc), benzene, toluene, THF, and [Emim^+^][NTf_2_^−^] were purchased from Wako Pure Chemicals.

*N*-[3-(Trimethoxysilyl)propyl]aniline, methyl methacrylate (MMA) 1,8-bis(dimethylamino)naphthalene, trioctylamine (TOA), copper(i) bromide, 2,2′-bipyridine, and trimethylamine were purchased from Sigma-Aldrich Co. LLC.

### Preparation of F

F were synthesized following the two steps reported by Yang *et al.*^47^ with some modification: hydrothermal synthesis of α-Fe_2_O_3_ nanoplates and wet chemical reduction of the α-Fe_2_O_3_ nanoplates.

In the first step, the synthetic procedure of α-Fe_2_O_3_ nanoplates followed the synthetic method proposed by Chen *et al.*^47,48^ First, 0.27 g of FeCl_3_·6H_2_O, 10 mL of ethanol, and 0.70 mL of distilled water were mixed with vigorous stirring. After dissolution, 0.80 g of sodium acetate was added into the mixing solution while stirring constantly. Then, the mixtures were transferred into a Teflon-lined autoclave and maintained at 180 °C for 12 h. After cooling the autoclave to room temperature, the resulting red particles were centrifuged and washed with ethanol and distilled water. Finally, α-Fe_2_O_3_ nanoplates were obtained by drying at 60 °C for 8 h.

In the second step, F were synthesized *via* wet chemical reduction of the resulting α-Fe_2_O_3_ nanoplates.^47^ In the wet chemical reduction, 0.10 g of α-Fe_2_O_3_ nanoplates and 25 mL of TOA were mixed in a three-neck flask with a reflux condenser under ultrasonic irradiation. Then, the mixtures were gradually heated to 450 °C and maintained at this temperature for 15 min under a sufficient flow of 3% H_2_/Ar. The colour of the mixtures changed from red to black when the reduction completely finished. In addition, 3% H_2_/Ar needed to be continuously flowed before cooling to room temperature. The resulting F were centrifuged and washed with THF and distilled water several times. Then, F were dried at 60 °C. To remove the residual organic chemicals on the surface of F, they were heated to 260 °C at a heating rate of 100 °C min^−1^ and maintained at this temperature for 1 min under N_2_ gas flow.

### Introduction of SI-ATRP initiators onto the surface of F

Before grafting PMMA chains onto the surface of F, *p*-(bromomethyl)benzyl 2-bromoisobutylate (BBI),^18^ as an SI-ATRP initiator, needed to be introduced onto the surface of F. Initially, 0.40 g of F was dispersed in 40 mL of ethanol and ultrasonicated for 1 h. Then, 0.16 mL of hexylamine and 0.40 mL of distilled water were added into the mixtures and dispersed under ultrasonic irradiation for 3 h. After adding 0.80 mL of *N*-[3-(trimethoxysilyl)propyl]aniline, the dispersion was treated by ultrasonic irradiation at 50 °C for 3 h to introduce aniline groups. Then, aniline-modified F were obtained after washing with acetonitrile and drying under vacuum.

In the next step, 0.060 g of 1,8-bis(dimethylamino)naphthalene (proton-sponge) and 0.40 g of aniline-modified F were dispersed in 15 mL of acetonitrile with 3 h of ultrasonic irradiation. Finally, 0.42 g of BBI as an SI-ATRP initiator was added into the mixtures at 40 °C with 12 h of ultrasonic irradiation. Then, the resulting F with SI-ATRP initiators were washed with methanol several times and dried under vacuum.

### Polymer modification *via* SI-ATRP

The preparation of the PMMA-modified F*via* SI-ATRP was performed as follows: 20 mg of initiator-modified F and 7.2 mg of copper(i) bromide were added into a vacuum-sealed tube. Then, 1.8 μL of benzyl 2-bromoisobutylate (BI) as a free initiator was dissolved in 6.0 mL of DMAc. Then, BI/DMAc solution was added to the vacuum-sealed tube, which was evacuated to avoid the inhibitory effect of oxygen. Then, the mixtures were treated by ultrasonic irradiation for 30 min. A total of 16 mg of 2,2′-bipyridine dissolved in 6.0 mL of MMA was added to the vacuum-sealed tube, which was evacuated in the same way. The mixtures were heated at 60 °C with stirring for 1 h of polymerization. Then, the mixtures were quenched, precipitated by methanol and collected by filtration. After drying at room temperature, the PMMA-modified F were centrifuged and washed with THF 3 times and benzene 1 time. The supernatant THF was collected and concentrated by an evaporator to obtain the pure PMMA chains grown from BI. The PMMA-modified F and pure PMMA chains were re-dispersed into benzene and frozen by liquid N_2_. Powder samples were obtained after 12 h of freeze-drying. According to the different polymerization times, the obtained PMMA-modified F were marked as FP1 (1 h), FP2 (6 h), and FP3 (12 h). The molecular weight of the corresponding pure PMMA chains was the same as the molecular weight of the PMMA chains on the surface of F, which was measured by size exclusion chromatography (SEC).

### Mixture of FP*m* and [Emim^+^][NTf_2_^−^]

To prepare the mixtures of FP*m* (*m* = 1, 2, 3) and [Emim^+^][NTf_2_^−^], FP*m* and [Emim^+^][NTf_2_^−^] were dissolved in THF to form mixing solutions with a concentration of 10 mg mL^−1^. Then, the mixing solutions, FP*m*/THF and [Emim^+^][NTf_2_^−^]/THF, were mixed at weight ratios of 3/1, 2/1, 1/1, 1/2, and 1/3 (FP*m*/[Emim^+^][NTf_2_^−^]) under ultrasonic irradiation. Finally, the final mixture samples were obtained by evaporating THF at 60 °C under Ar gas flow. Based on observations of the obtained mixture samples with different weight ratios from 3/1 to 1/3, FP*m* and [Emim^+^][NTf_2_^−^] mixed uniformly without any phase separation.

### Characterization

X-ray diffraction (XRD) measurements were performed on a Rigaku Ultima-IV system using Cu Kα radiation at 40 kV and 40 mA. In addition, the diffraction patterns were recorded in 2*θ* over the range of 10–80°. Transmission electron microscopy (TEM) observations were performed using a HITACHI H-7650 with an acceleration voltage of 100 kV. High-resolution TEM (HR-TEM) and scanning transmission electron microscopy (STEM) images were obtained on a Titan^3^™ 60-300 Probe Corrector (FEI-Company) at an acceleration voltage of 300 kV. SEC analysis was carried out at 40 °C using a Tosoh HLC-8220 with guard columns of TSKgel Guard Column SuperHZ-L and a set of four separation columns of TSK-gel SuperHZ4000, TSKgel SuperHZ3000, TSKgel SuperHZ2000, and TSKgel SuperHZ1000. THF was used as the eluent at a flow rate of 0.35 mL min^−1^. The SEC system was calibrated with standard polystyrene samples (Tosoh, PStQuick). The specific surface areas of particles were determined using nitrogen adsorption isotherms obtained with a BEL Japan, Inc. BELSORP-MINI. All samples were treated with heat at 120 °C for 30 min under N_2_ flow before measurement. Thermogravimetric analysis (TA) was performed using a Rigaku Thermo plus EVO2. Thermal characterization was carried out with a TA Instruments MDSC Q-100 equipped with an electric cooling system. Fourier transform infrared spectroscopic (FT-IR) observation was performed by an IRAffinity-1S (SHIMADZU) device. Ultra-small angle X-ray scattering (USAXS) measurements were carried out at SPring-8 BL03XU equipped with a PILATUS 1M detector (Dectris®). A KEYENCE VHX-2000 equipped with a Mettler FP82 HT hot stage was utilized to take polarized optical microscopy (POM) images. The magnetic property was measured by a superconducting quantum interference device (SQUID) magnetometer (Quantum Design, MPMS series) at room temperature (300 K).

## Results and discussion

**Fig. 1:**
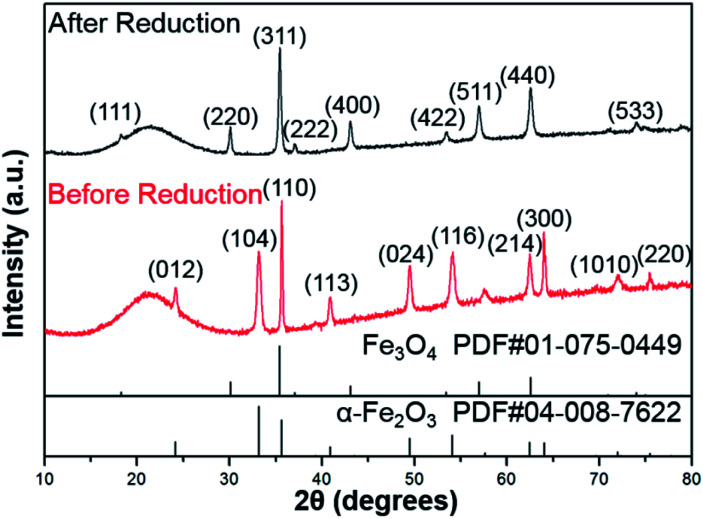
XRD profiles of α-Fe_2_O_3_ (before reduction) and F (after reduction).

**Fig. 2:**
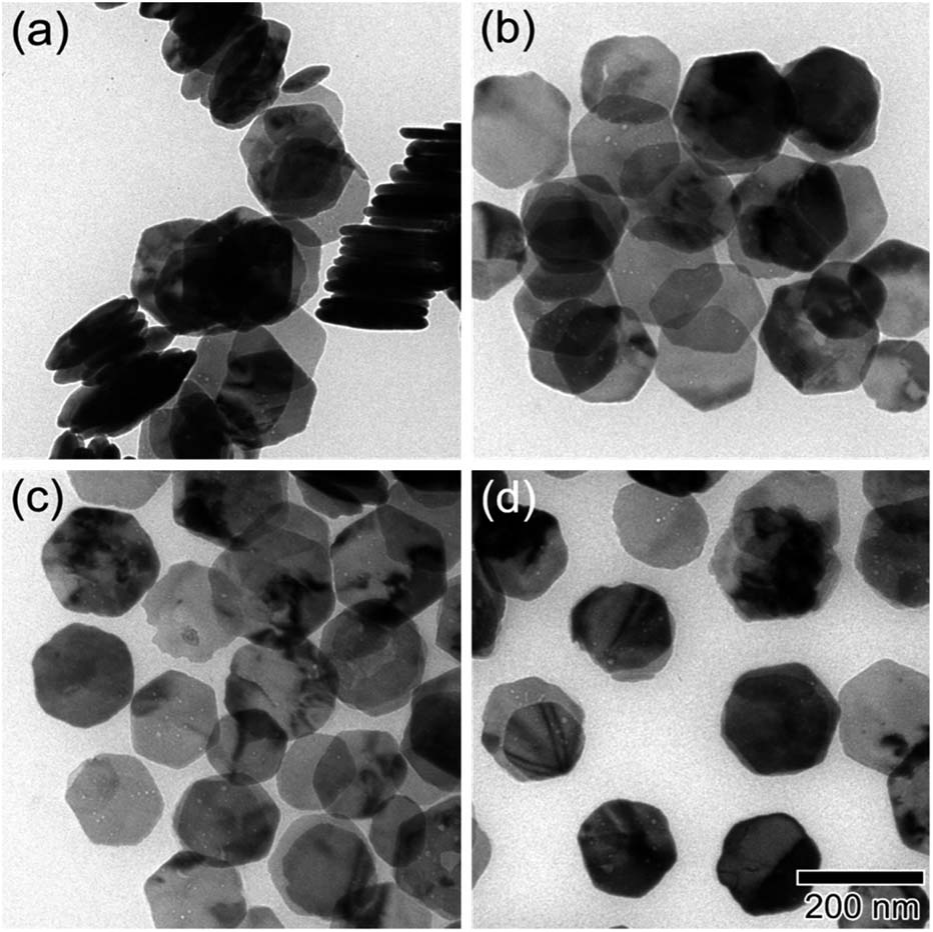
TEM images of (a) F, (b) FP1, (c) FP2, and (d) FP3. The scale bar shown in (d) is common to all images.

As shown in Fig. 1, the XRD profiles illustrate that α-Fe_2_O_3_ was successfully reduced to single phase Fe_3_O_4_. After being modified by PMMA chains *via* SI-ATRP, FP*m* were dispersed in toluene under ultrasonic irradiation and then cast on TEM grids. Representative TEM images of F, FP1, FP2, and FP3 are shown in Fig. 2. As shown in Fig. 2a, uniform hexagonal plates and slender sides can be clearly observed for F. The average size and thickness of F are 176.5 ± 18.7 nm and 23.3 ± 2.9 nm, respectively (aspect ratio: 7.7). In addition, the PMMA-unmodified F attracted each other due to magnetic attractive forces. From Fig. 2b–d, it can be observed that FP*m* could not easily overlap with each other because of the existence of thick PMMA chains on the surface of F. Furthermore, the distance between FP*m* obviously increased with increasing polymerization time. Therefore, the introduction of PMMA chains effectively inhibited the aggregation of FP*m*.

To study the effect of the molecular weight of PMMA chains on the various properties, the number average molecular weight (*M*_n_) and weight average molecular weight (*M*_w_) of PMMA were determined by SEC (see Experimental section). The *M*_n_ of PMMA chains on FP1, FP2 and FP3 was 3.2 × 10^4^, 6.7 × 10^4^, and 9.2 × 10^4^, respectively. The *M*_w_ of PMMA chains on FP1, FP2, and FP3 was 3.7 × 10^4^, 8.5 × 10^4^, and 1.3 × 10^5^, respectively. The results of SEC measurements indicate that the PMMA chain length increased with increasing polymerization time. More information, such as the ATRP initiator modification density (*D*_I_), PMMA modification density (*D*_P_), weight fraction (*F*_w_), volume fraction (*F*_v_), FT-IR spectra and TA data, is summarized in the ESI.† According to the *D*_P_ and the average surface area of individual nanoplates (3.3 × 10^4^ nm^2^), the number of PMMA chains grafted on the surface of FP1, FP2, and FP3 was calculated to be 5600, 8600, and 12 000, respectively.

POM observations were utilized to check the lyotropic LC phases of FP*m* in [Emim^+^][NTf_2_^−^]. Optical birefringence could be observed in the POM images of FP3/[Emim^+^][NTf_2_^−^] (weight ratio: 1/3) due to the formation of lyotropic LC phases even when it was heated to 200 °C (see Fig. S4†).

**Fig. 3:**
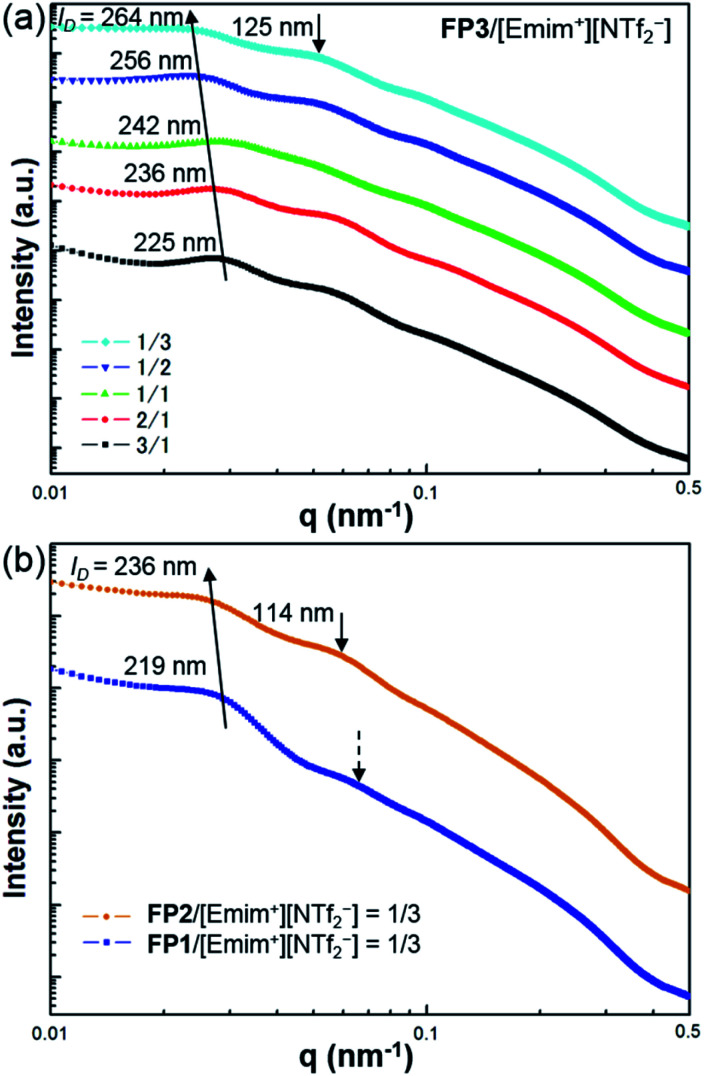
USAXS curves of (a) FP3/[Emim^+^][NTf_2_^−^] with different weight ratios, 3/1, 2/1, 1/1, 1/2, and 1/3, and (b) FP1/[Emim^+^][NTf_2_^−^] and FP2/[Emim^+^][NTf_2_^−^] at the weight ratio of 1/3. The measurements were carried out at SPring-8 BL03XU at 25 °C.

The self-organized structures of the lyotropic LC phases of FP*m*/[Emim^+^][NTf_2_^−^] were investigated by USAXS measurements at SPring-8 BL03XU. USAXS images were taken with the PILATUS 1M detector (Dectris®). Then, the USAXS images of FP1/[Emim^+^][NTf_2_^−^], FP2/[Emim^+^][NTf_2_^−^], and FP3/[Emim^+^][NTf_2_^−^] were converted into USAXS curves (intensity-*q* patterns), as shown in Fig. 3.

The average distance between FP*m* is an important influential factor for the scattering peaks, which is defined as the interparticle distance (*I*_D_). The *I*_D_ between FP*m* can be determined and calculated from USAXS measurements according to Bragg's law. As shown in Fig. 3a, the corresponding *I*_D_ values of FP3/[Emim^+^][NTf_2_^−^] with weight ratios of 3/1, 2/1, 1/1, 1/2, and 1/3 are marked on the USAXS curves. Based on a comparison of the different weight ratio FP3/[Emim^+^][NTf_2_^−^], *I*_D_ changed from 225 nm to 264 nm over the wide range of weight ratios from 3/1 to 1/3. This indicates that changing the weight ratio of [Emim^+^][NTf_2_^−^] is an effective means of controlling the *I*_D_ values. The same regularity could also be found in the USAXS curves of FP1/[Emim^+^][NTf_2_^−^] and FP2/[Emim^+^][NTf_2_^−^]. In addition, the results of USAXS measurements show that *I*_D_ could be increased by increasing the PMMA chain length. Take the weight ratio of 1/3 (Fig. 3b) for instance. The *I*_D_ of FP3/[Emim^+^][NTf_2_^−^] (264 nm) was wider than those of FP1/[Emim^+^][NTf_2_^−^] (219 nm) and FP2/[Emim^+^][NTf_2_^−^] (236 nm) at the weight ratio of 1/3, which is consistent with TEM observations of FP*m*. Hence, SI-ATRP realized effective control of the *I*_D_ of FP*m*/[Emim^+^][NTf_2_^−^] in the lyotropic LC systems. The results also suggest that FP*m* would show higher miscibility in [Emim^+^][NTf_2_^−^] when increasing the PMMA chain length. In particular, secondary peaks could be obviously observed on the USAXS curves when the weight ratio of [Emim^+^][NTf_2_^−^] was low (FP3/[Emim^+^][NTf_2_^−^]: 3/1 and 2/1). When the weight ratio of [Emim^+^][NTf_2_^−^] was high, no obvious secondary peaks could be observed (see dashed arrows) on the USAXS curves of FP1/[Emim^+^][NTf_2_^−^] (weight ratio: 1/3). The low orientational order of FP1/[Emim^+^][NTf_2_^−^] (weight ratio: 1/3) was thought to be the main reason for the weakness of the scattering intensity. The obvious secondary peaks (marked as black arrows) on the USAXS curves of FP2/[Emim^+^][NTf_2_^−^] and FP3/[Emim^+^][NTf_2_^−^] (weight ratio: 1/3) demonstrate an increase in the orientational order due to the growth of PMMA chains. Hence, the above results indicate that FP3 exhibited good lyotropic LC behaviour and good dispersion stability in [Emim^+^][NTf_2_^−^].

**Fig. 4:**
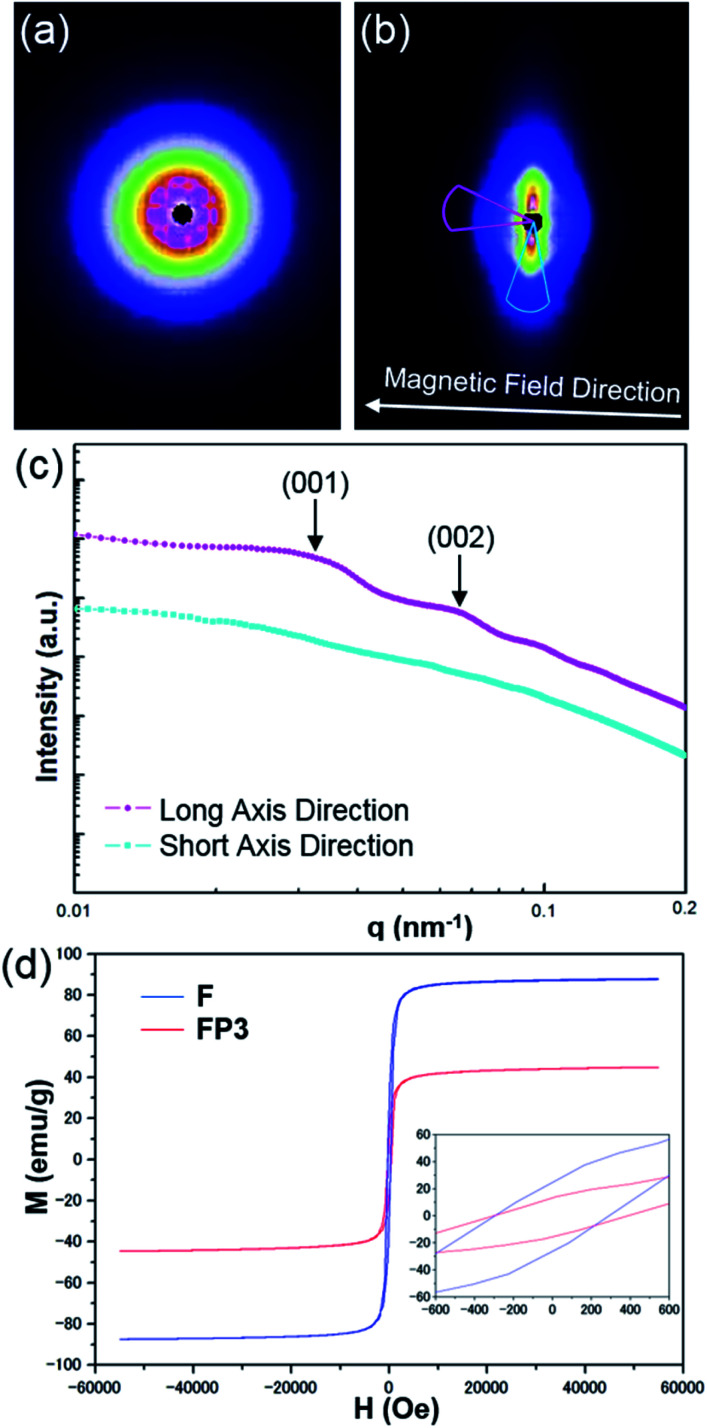
USAXS images of FP3/[Emim^+^][NTf_2_^−^] (weight ratio: 1/3) (a) without an external magnetic field and (b) with an external magnetic field (320 Oe), (c) USAXS curves of the long and short axes of the elliptic scattering image at 320 Oe, and (d) hysteresis loops of F and FP3 at room temperature (300 K).

A magnetic field can also significantly affect the formation of LC structures. To study the LC structures and their responsiveness to the presence of an external magnetic field, FP3/[Emim^+^][NTf_2_^−^] (weight ratio: 1/3) mixtures were studied based on USAXS measurements while applying an external magnetic field at 25 °C using custom-made 2-axis magnetic field equipment. The magnetic field strength was adjusted to 320 Oe. The USAXS images of FP3/[Emim^+^][NTf_2_^−^] (weight ratio: 1/3) without the external magnetic field and with the external magnetic field are shown in Fig. 4a and b, respectively. The scattering images show that the isotropic circular scattering image changed to an anisotropic elliptic scattering image in a split second after applying the external magnetic field because of the fast response of the lyotropic LCs to the external magnetic field. The direction of the external magnetic field is vertical to the short axis of the elliptic scattering image (Fig. 4b). As shown in Fig. 4c, the short axis (pale blue region) and long axis (pink region) of the anisotropic elliptic scattering image were converted into intensity-*q* patterns with a scanning range of 30°. Two broad scattering peaks were observed on the long axis curve, while no broad scattering peaks were observed on the short axis curve. The results revealed that FP3/[Emim^+^][NTf_2_^−^] maintained a uniaxial alignment structure under the external magnetic field. According to the two broad scattering peaks in the long axis direction, the corresponding *d* values could be indexed to 195 nm and 95 nm, respectively. These two peaks were assigned to the (001) and (002) planes of the columnar structure formed by the stacking of FP3 in [Emim^+^][NTf_2_^−^] under the external magnetic field (the detailed columnar structure is discussed below). On the other hand, the *I*_D_ of FP3/[Emim^+^][NTf_2_^−^] (weight ratio: 1/3) was 264 nm before application of the external magnetic field. Obviously, *I*_D_ shrunk under the action of the external magnetic field. Generally, stimulated LCs with a polydomain structure form a uniaxial alignment along the magnetic field direction while maintaining the interparticle distance.^30^ However, the *I*_D_ of FP3/[Emim^+^][NTf_2_^−^] decreased in this system after applying an external magnetic field. The most likely reason is that the dynamic control of *I*_D_ was affected by the function of the soft PMMA chains, and the self-organized structure of the lyotropic LCs changed due to the external stimulation of the magnetic field. The same magnetic responsiveness was also observed in FP1/[Emim^+^][NTf_2_^−^] and FP2/[Emim^+^][NTf_2_^−^]. The results indicate that applying an external magnetic field is also a feasible means to control *I*_D_. To further study the effect of PMMA chains on the magnetic behaviour of F, magnetization of F and FP3 was evaluated by a SQUID system with an applied magnetic field of 55 kOe. Fig. 4d shows the hysteresis loops of F and FP3 at room temperature (300 K). The saturation magnetization (*M*_s_) of F and FP3 was approximately 87.8 emu g^−1^ and 44.8 emu g^−1^, respectively. From the hysteresis loops, it can be seen that F and FP3 can exhibit ferromagnetic behaviour. The existence of PMMA chains increased the surface spins disorientation, which resulted in the decline of *M*_s_ values.

**Fig. 5:**
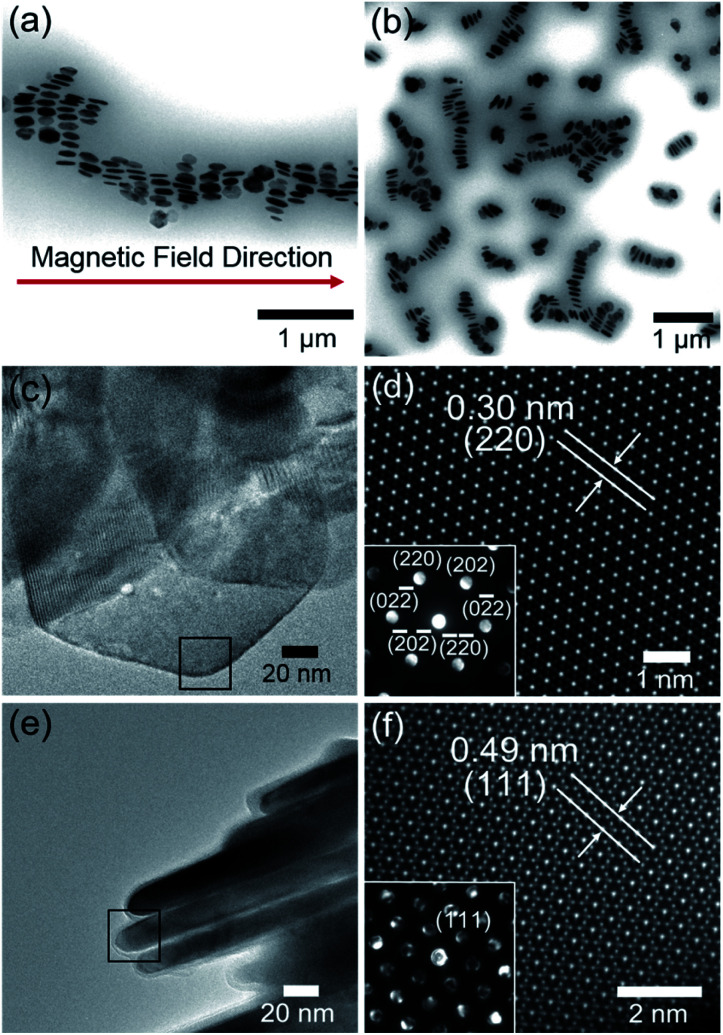
(a) TEM image of FP3 under a parallel magnetic field, (b) TEM image of FP3 under a vertical magnetic field, (c) HR-TEM image of horizontally lying F, (d) STEM image and SAED image (inset) of the selected black area in (c), (e) HR-TEM image of vertically standing F, and (f) STEM image and SAED (inset) image of the selected black area in (e).

To investigate the plausible self-organized structure of the lyotropic LC phases, a drop of FP3/toluene solution (concentration: 0.1 g L^−1^) was cast on a TEM grid after applying an external magnetic field vertical or parallel to the TEM grid, as shown in Fig. S5.†Fig. 5a shows a TEM image of FP3 under a parallel magnetic field. Astonishingly, it was observed that FP3 were simultaneously stacked on the TEM grid and aligned in the magnetic field direction, which was a structure closer to a nematic columnar LC phase than to a discotic nematic LC phase.^28^ A TEM image of FP3 under a vertical magnetic field is shown in Fig. 5b. Most of the FP3 were standing in a columnar structure on the TEM grid under the action of the vertical magnetic field. The interparticle distance between two aligned nanoplates was approximately 190 nm. The results give us some hints about the alignment of FP3 in organic solvents (such as [Emim^+^][NTf_2_^−^]) under an external magnetic field. To explore the mechanism of the special alignment of nanoplates, HR-TEM, STEM, and selected area electron diffraction (SAED) were utilized to analyse the growth axis of F. The STEM image shown in Fig. 5d was taken from the selected black area of the horizontally lying F in Fig. 5c. Its lattice spacing was approximately 0.30 nm, which corresponds to the (220) plane of F. The corresponding SAED pattern is also shown in Fig. 5d. The six-fold diffraction spots in the SAED pattern could be assigned to the equivalent (220) planes of F. The six equivalent planes were all parallel to the [111] direction. Hence, the top and bottom surfaces of F grew along the [111] direction. Similarly, vertically standing F were also observed by HR-TEM, STEM, and SAED, as shown in Fig. 5e and f. The corresponding lattice spacing of the slender sides was approximately 0.49 nm, which could be indexed to the (111) plane of F. Therefore, the growth axis of F was along the [111] direction.

It is well known that the easy magnetic axes of Fe_3_O_4_ are along the [110] and [111] directions, where the [111] direction is an easier magnetic direction than the [110] direction. Generally, most of the F should tend to lie vertically along the magnetic field direction, as shown in Fig. S6.† However, the PMMA-modified F tended to stand and arrange along the magnetic field direction. Regarding the reason for this, the agglomeration of PMMA-modified F might decrease the possibility of the exposure of the [111] plane, resulting in the penetration of the magnetic field from the slender sides. Zhou *et al.*^49^ mentioned that the agglomeration may produce stronger dipolar fields than the individual nanoplates. Magnetic dipole with the marked directivity would exist in the center of magnetic coil. The axial direction of dipolar fields is same with the normal direction of magnetic coil. It is relatively difficult for an external magnetic field to penetrate the axial direction of dipolar fields, while [111] plane is parallel to the axial direction. In other words, stronger dipolar fields between PMMA-modified F would decrease the possibility of the exposure of the [111] plane. In fact, there are also excluded volume effects for parallel plate particles. It was reported by Onsager^50^ that the force (per unit area) between two parallel plates exponentially decreases in an electric field with increasing distance between the parallel plates because of the excluded volume effects. In other words, plates that are kept away from each other would block the electromagnetic effect due to the decrease in the force (per unit area) between the two parallel plates. Such a result would inevitably block the exposure of the [111] plane, providing opportunities for the [110] plane to be exposed to a magnetic field. These reasons explain why it is difficult for a magnetic field to penetrate the front side of FP3, although the [111] direction is the easy magnetic axis. Hence, the interparticle distance of FP3 caused by the complicated entanglement of PMMA chains would block the exposure of the [111] plane, resulting in the uniaxially aligned nematic columnar structure of FP3 along the magnetic field direction. The above results demonstrate that PMMA-modified F are affected by the controllable interparticle interaction even under an external magnetic field.

**Fig. 6:**
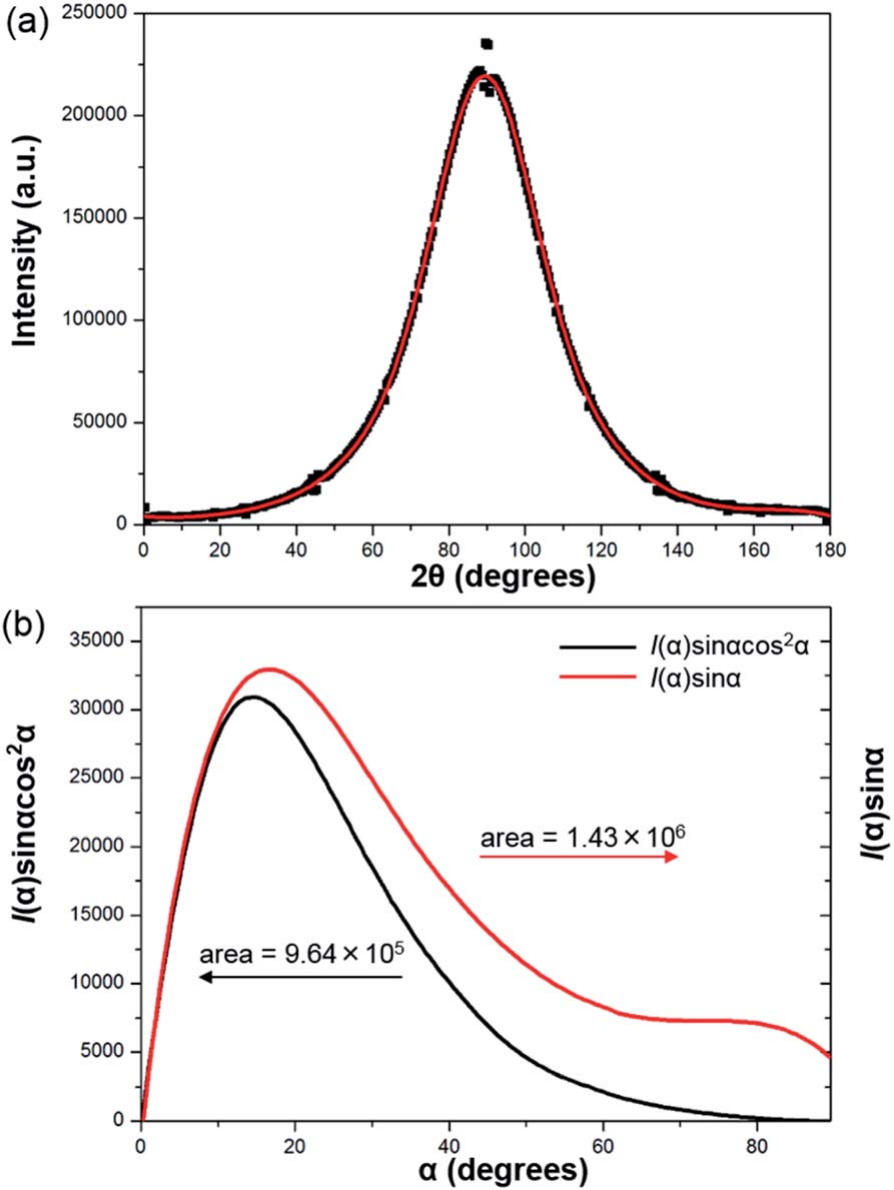
(a) Azimuthal intensity distribution *I*(*χ*) of FP3/[Emim^+^][NTf_2_^−^] (weight ratio: 1/3) under an external magnetic field at 2*θ* = 0.5°, with the red line a linear fit, and (b) graphical presentation of the two integrals in the ratio that determine 〈cos^2^ *α*〉.

To further prove the formation of lyotropic LC phases, the orientational order parameter (*S*) was also investigated by USAXS measurement. As shown in Fig. 6a, the azimuthal intensity distribution *I*(*χ*) of FP3/[Emim^+^][NTf_2_^−^] (weight ratio: 1/3) under an external magnetic field was evaluated at 2*θ* = 0.5° with a linear fit. Then, the order parameter *S* was calculated by referring to the existing formulas.^51,52^
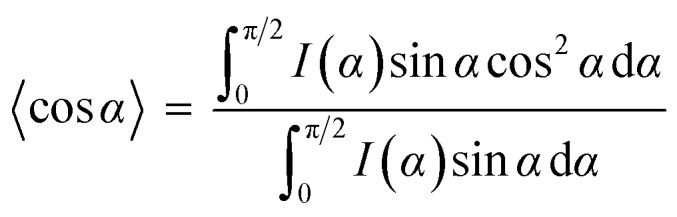

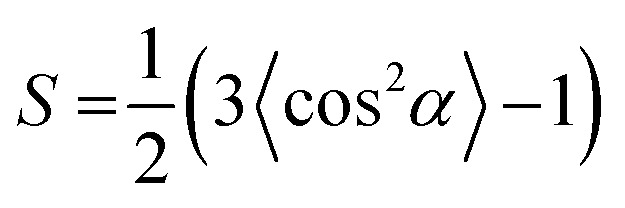
where *I* is the USAXS intensity and *α* is the angle of the nematic columnar plane of the lyotropic LC domains under the external magnetic field. The intensity distribution in the samples *I*(*α*) was determined from the azimuthal intensity *I*(*χ*) by cos *α* = cos *χ* cos *θ*, where *θ* expresses the Bragg angle. The transformation results could provide intensity values over the entire range (*α* = 0° to 90°). The angle at the maximum peak intensity was set as *α* = 0°. Fig. 6b shows a graphical presentation of *I*(*α*) sin *α* cos^2^ *α* and *I*(*α*) sin *α*, which were used to determine 〈cos^2^ *α*〉 from the ratio of the integral areas under the red and black lines. The order parameter *S* could be calculated as 0.51. Hence, the above results indicate that FP3 exhibited a nematic columnar structure in [Emim^+^][NTf_2_^−^] under an external magnetic field.

**Fig. 7:**
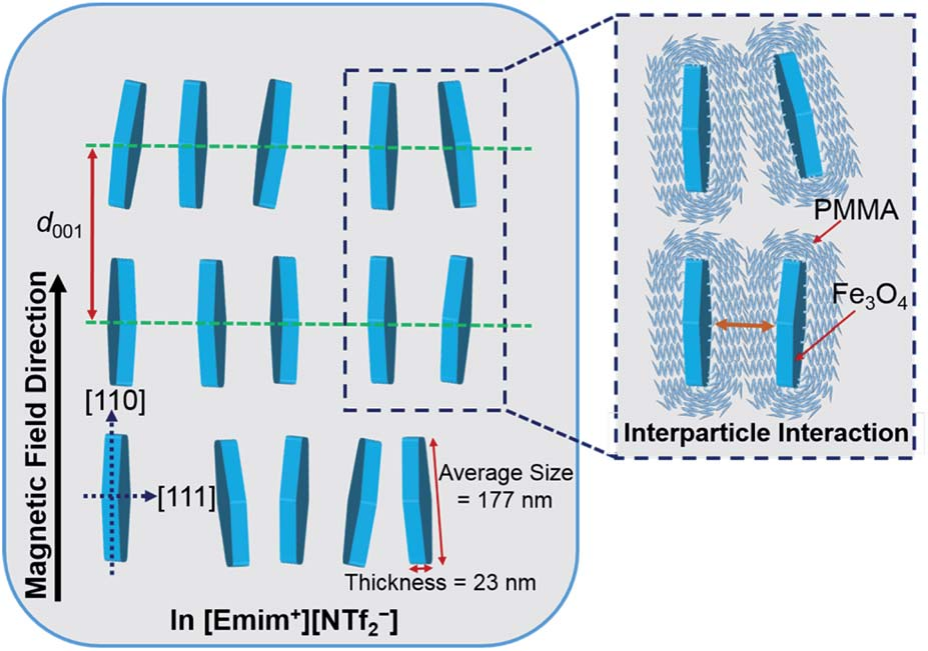
The most plausible model of the magnetic field inducing the lyotropic nematic LC self-organized structure of FP3/[Emim^+^][NTf_2_^−^] (weight ratio: 1/3) with controllable interparticle interaction.

Based on the above analysis, the most plausible model of the magnetic field inducing the lyotropic nematic LC self-organized structure of FP3/[Emim^+^][NTf_2_^−^] (weight ratio: 1/3) with controllable interparticle interaction was predicted and is shown in Fig. 7. All of the nanoplates are PMMA-modified F. The enlarged image shows the PMMA chains on the surface of F. The average size (177 nm) and thickness (23 nm) of F were determined by TEM observation. *d*_001_ refers to the spacing of the nematic columnar structure corresponding to the long axis of the elliptic scattering image at 320 Oe. The magnetic field direction is parallel to the [110] direction but vertical to the [111] direction of the Fe_3_O_4_ crystal. Fig. 7 also illustrates the synergistic effect of the external magnetic field and interparticle interaction on FP3/[Emim^+^][NTf_2_^−^]. Such a magnetic field responsive model for the special alignment of PMMA-modified F with controllable interparticle interaction has not been reported thus far.

## Conclusions

In summary, F were synthesized through wet chemical reduction of α-Fe_2_O_3_ nanoplates, which was verified by the XRD results. Afterwards, F were modified by PMMA *via* SI-ATRP after introducing SI-ATRP initiators. PMMA chains played a positive role in effectively preventing the aggregation of F. By increasing the PMMA chain length, the distance between FP*m* was obviously increased in common organic solvents. In addition, FP3 exhibited good dispersion stability for a wide range of weight ratios and lyotropic LC properties in [Emim^+^][NTf_2_^−^] (weight ratio of FP3/[Emim^+^][NTf_2_^−^]: 1/3). Both the PMMA chain length and the mixing weight ratio have significant effects on the *I*_D_ values. It is important to note that application of an external magnetic field can lead to a rapid LC respond and shorten *I*_D_. In addition, the lyotropic LCs could form nematic phases with a columnar alignment under an external magnetic field. We also identified the mechanism of the special alignment of FP3 under the external magnetic field. Because the *I*_D_ of FP3 blocked the exposure of the [111] plane due to the long PMMA chains, FP3 with controllable interparticle interaction maintained a uniaxially aligned nematic columnar structure along the magnetic field direction. The magnetic field responsive behaviour for the special alignment of PMMA-modified F with controllable interparticle interaction might have vast research potential for functional fluids. They can be expected to help improve the performance of MR fluids, magnetic fluids, and lyotropic LCs. Furthermore, this research might be closely connected with the design of new devices, such as rewritable magnetic switching devices and smart magneto-electrochemical nanosensors.

## Conflicts of interest

There are no conflicts to declare.

## Supplementary Material

NA-002-C9NA00767A-s001
