# Supplementary material for: Magnetic field induced uniaxial alignment of the lyotropic liquid-crystalline PMMA-grafted Fe3O4 nanoplates with controllable interparticle interaction
Source: Nanoscale Adv. 2020 Jan 14;2(2):814–22. doi: 10.1039/c9na00767a (PMC9417206; doi:10.1039/c9na00767a)
Supplement: NA-002-C9NA00767A-s001 [file NA-002-C9NA00767A-s001.pdf]

Electronic Supplementary Material (ESI) for Nanoscale Advances.
This journal is © The Royal Society of Chemistry 2020

(Supplementary Information)

Magnetic field induced uniaxial alignment of the lyotropic liquid-crystalline PMMA-grafted Fe_3O_4 nanoplates with controllable interparticle interaction

Chen Shen,[a] Masaki Matsubara,[b] Mizuho Yabushita,[a] Sachiko Maki,[a] Atsushi Muramatsu,[a] Kiyoshi Kanie*[a]

[a]*Institute of Multidisciplinary Research for Advanced Material, Tohoku University, 2-1-1 Katahira, Aoba-ku, Sendai, Miyagi 980-8577, Japan.*

[b]*National Institute of Technology, Sendai College, 48 Nodayama, Medeshima-Shiote, Natori, Miyagi 981-1239, Japan.*

(E-mail: kanie@tohoku.ac.jp)

1. Determination of modification densities on Fe_3O_4

1.1 Calculation of modification densities of amine groups and SI-ATRP initiators on Fe_3O_4

Modification amounts of amine groups and SI-ATRP initiators on **F** were determined by TA. The TA measurements were carried out under Ar gas, and the heating ratio was fixed to 10 ºC/min. Here, amine-groups modified **F** and SI-ATRP initiator-modified **F** were abbreviated by **FN** and **F***, respectively. Weight losses of **F**, **FN**, and **F*** by TA measurements were abbreviated as L_F (%), L_{FN} (%), and L_{F*} (%), respectively. The modification density of amine groups on **F** was calculated by the following equation (eq. S1). Here, N_A is Avogadro constant and SA_{ES} is the estimated specific surface area of **F**. The measurement result of SA_{ES} is 14 m^2/g. MW_a means a molecular weight of organic moieties on **F**. In this case, we subtracted molecular weight of trimethoxysilyl group from molecular weight of *N*-[3-(trimethoxysilyl)propyl]aniline to calculate MW_a (MW_a = 134).

$$\text{Amine-groups modification density} = \frac{N_A\left(\frac{L_{FN}}{100-L_{FN}} - \frac{L_F}{100-L_F}\right)}{SA_{ES}\times MW_a}\ chains/nm^2 \qquad \text{eq. S1}$$

Next, SI-ATRP initiator modification densities (D_I) on **F** were calculated by the following equation (eq. S2 and S3). Here, x means reaction rate of amine groups in the initiator modification and y means amine groups modification amount for 1 g of **F**. MW_b means a molecular weight of organic moieties on **F** which subtracted molecular weight of trimethoxysilyl group after reacting with BBI (MW_b = 401).

$$x\cdot y\cdot MW_b + (1-x)\cdot y\cdot MW_a = \frac{L_{F*}}{100-L_{F*}} - \frac{L_F}{100-L_F}$$

$$y = \frac{\left(\frac{L_{FN}}{100-L_{FN}} - \frac{L_F}{100-L_F}\right)}{MW_a}$$

$$x = \frac{\frac{L_{F*}}{100 - L_{F*}} - \frac{L_F}{100 - L_F} - y \cdot MW_a}{y \cdot (MW_b - MW_a)} \qquad \text{eq. S2}$$

$$D_I = x \cdot y \cdot \frac{N_A}{SA_{ES}} chains / nm^2 \qquad \text{eq. S3}$$

Fig. S1 shows TA profiles of **F**, amine-groups modified **F**, and SI-ATRP initiator-modified **F**.

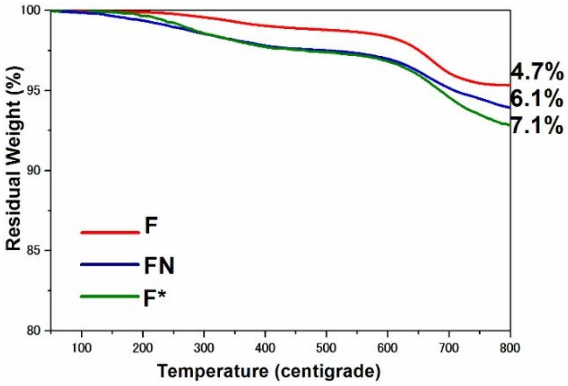

Fig. S1 TA profiles of **F**, **FN** and **F***.

1.2 PMMA modification densities

Modification amounts of PMMA were also determined by TA. Weight of PMMA for 1 g of **F** was marked as W_{FP}. Here, L_{FP}% is weight loss of **FP*m*** (***m*** = 1, 2, 3).

$$W_{FP} = \frac{L_{FP}}{100 - L_{FP}} - \frac{L_{F*}}{100 - L_{F*}} - \frac{L_F}{100 - L_F} \qquad \text{eq. S4}$$

Next, PMMA modification densities (D_P) were calculated by the following equation (eq. S5). *Mn* means number average molecular weight of PMMA on **F**, which was determined by SEC.

$$D_P = \frac{W_{FP}}{Mn} \cdot N_A \cdot \frac{1}{SA_{ES}} chains / nm^2 \qquad \text{eq. S5}$$

Fig. S2 shows TA profiles of **FP*m***.

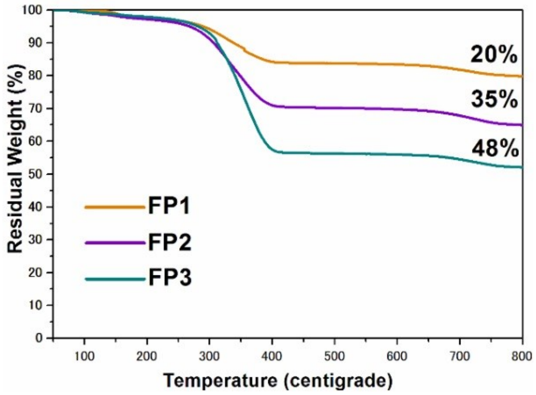

Fig. S2 TA profiles of **FP*m***.

1.3 Calculation results

The D_I values, D_P values and molecular weight distribution were listed in Table. S1. The weight fractions (*Fw*) and volume fractions (*Fv*) mean the proportion of PMMA chains' weight and volume in overall **FP*m***, respectively.

Table. S1 Characteristics of PMMA-modified **F**: **FP1**, **FP2** and **FP3**.

	Mw/Mn	D_I[a] (chains/nm^2)	D_P[b] (chains/nm^2)	*Fw*[c]	*Fv*[d]
**FP1**	1.17		0.17	0.11	0.35
**FP2**	1.27	1.85	0.26	0.29	0.64
**FP3**	1.43		0.37	0.44	0.77

[a]Modification densities of SI-ATRP initiator, [b]modification densities of PMMA, [c]weight fraction of PMMA and [d]volume fraction of PMMA.

2. FT-IR

Fig. S3 shows the FT-IR spectra of **F**, **FP3**, and PMMA. In the spectra of **FP3** as shown in Fig. S3b, the peaks at 2999 cm^{-1} and 2947 cm^{-1} are assigned to the C-H bond, and the peaks at 1724 cm^{-1} are attributed to the C=O bond of PMMA. The FT-IR results indicate that large quantities of PMMA were grafted on the surface of **F**.

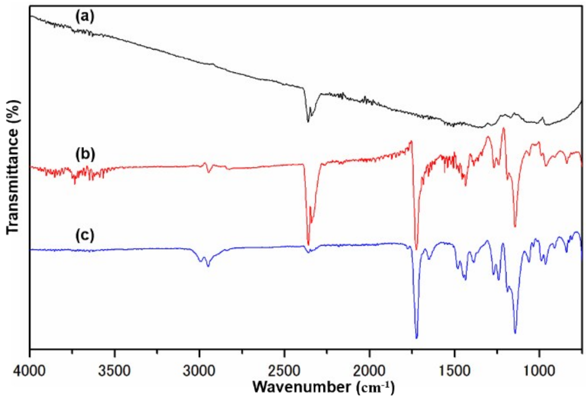

Fig. S3 FT-IR spectra of (a) **F**, (b) **FP3**, and (c) PMMA.

3. POM

POM observation was utilized to observe the lyotropic LC phases of **FP*m*** in ionic liquids. As is shown in Fig. S4a, only black images were observed because of the color of **F** and the not long enough PMMA chains. Fig. S4b exhibit the POM images of **FP3**/[Emim$^+$][NTf$_2^-$] (weight ratio: 1/3) at 200 ºC. Optical birefringence could be observed due to the formation of the lyotropic nematic phases even though were heated to 200 ºC from room temperature. However, obvious birefringence was not observed in other **FP3**/[Emim$^+$][NTf$_2^-$].

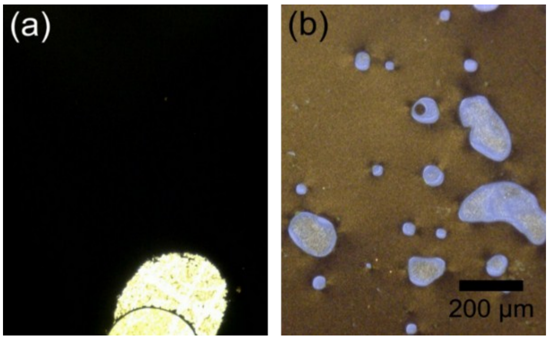

Fig. S4 POM images of (a) **FP1**/[Emim$^+$][NTf$_2^-$] (weight ratio: 1/2) , (b) **FP3**/[Emim$^+$][NTf$_2^-$] (weight ratio: 1/3) at 100 ºC. The scale bar shown in (b) is common for both of the two images.

4. Sketches of dripping FP3/toluene solution under magnetic field

A drop of **FP3**/toluene solution (concentration: 0.1 g/L) was dripped on a TEM grid after applying an external magnetic field that was vertical or parallel to the TEM grid.

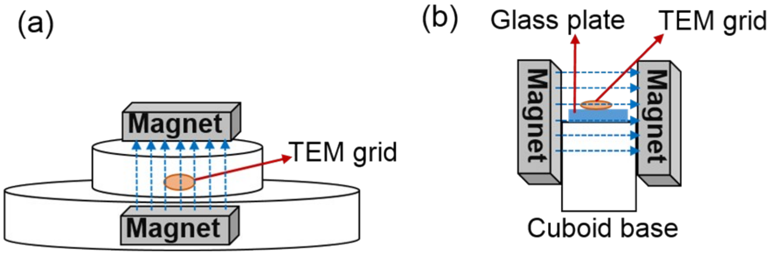

Fig. S5 Sketches of dripping **FP3**/toluene solution under a vertical magnetic field (a), or a parallel magnetic field (b).

5. TEM image of F under a vertical magnetic field

A drop of **F**/toluene solution (concentration: 0.05 g/L) was dripped on a TEM grid after applying an external magnetic field that was vertical to the TEM grid. Most of **F** lay vertically along the magnetic field direction as is shown in Fig. S6.

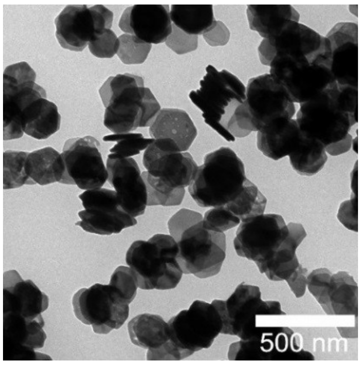

Fig. S6 TEM image of **F** under a vertical magnetic field.

6. Interparticle distance of FP*m* under an external magnetic field.

The interparticle distance results of **FP1**, **FP2**, and **FP3** under an external magnetic field (320 Oe), obtained from the USAXS curves, were listed in the Table. S2.

Table. S2 Interparticle distance results of **FP1**, **FP2**, and **FP3** under an external magnetic field (320 Oe).

	Interparticle distance (nm)
**FP1**	180
**FP2**	184
**FP3**	195
